# Wire Arc Additive Manufacturing: A Study of Process Parameters Using Multiphysics Simulations

**DOI:** 10.3390/ma16237267

**Published:** 2023-11-22

**Authors:** You Sung Han

**Affiliations:** Department of Mechatronics Engineering, Incheon National University, 119 Academy-ro, Yeonsu-gu, Incheon 22012, Republic of Korea; yshan@inu.ac.kr

**Keywords:** residual stress, finite element analysis, phase transformation, wire arc additive manufacturing

## Abstract

In this work, analyses focus on understanding the effects of the scanning pattern and speed on the thermal profile, phase transformation, and residual stress generation in the WAAM deposition. An FE numerical model is constructed that takes into account the phase evolution and transformation plasticity using the ABAQUS user subroutine, UMAT. The results show that the scanning pattern significantly affects the heat accumulation and the cooling rate during the AM deposition, and, eventually, the generation of residual stresses. According to the simulation results, the highest residual stress is generated in the case of the out–in scanning, while the alternate pattern leads to the lowest residual stress. The influence of the scanning speed on the thermal profiles and residual stress are also examined. The analyses show that an increase in the scan speed leads to a decrease in the peak temperature and an increase in the cooling rate, which result in an increase in the martensite volume fraction of the deposition.

## 1. Introduction

Additive manufacturing (AM) has gained significant attention due to its promising advantages. It allows for the production of net-shaped parts and structures with complex geometries in a remarkably reduced time. Metal AM technology has been employed in rapid prototyping, maintenance, and versatile manufacturing. Among the several methods of AM, wire arc additive manufacturing (WAAM) has been recognized as an emerging technology in many industries [1]. In WAAM, a high level of heat produced by a heat source is applied to a solid wire for the deposition. The deposition process involves multiphysics phenomena, namely interactions between the thermal, metallurgical, and mechanical behaviors of a material. A wide range of materials have been studied for applications in WAAM, including dissimilar steel-Al [2], titanium [3], aluminum [4,5], and others. During the WAAM process, the metal undergoes a phase transformation which results from an inhomogeneous thermal distribution throughout the process. The AM process parameters, such as the scan speed and pattern, play crucial roles in the thermal history of the AM part and induce microstructural changes [6,7,8]. These microstructural features can significantly affect the material’s properties and eventually the performance of the additive manufactured parts, including residual stress [9,10,11].

Gong et al. [12] studied the effects of beam scanning speed on the microstructures of Ti-6Al-4V in powder–beam electron beam additive manufacturing. They found that the size of the grain overall decreases as the scanning speed increases. Ravoori et al. [13] investigated the effects of the scan speed on the bonding of adjacent filaments in polymer extrusion-based manufacturing. The high-speed imaging analysis suggests that the raster scanning speed significantly affects the heat transfer and the bonding of the underlying and adjacent filaments. Wang and Chou [14] performed an EBSD study to investigate the effects of scan speed on the evolution of the microstructure and texture of a Ti-6Al-4V alloy. It was found that the intensity of the texture decreases as the scan speed increases. This finding implies that fine grain sizes and weak texture intensity result in high values of elastic modulus and hardness. Liu et al. [15] studied the evolution of the mechanical performance (i.e., microhardness and tensile strength) of selective laser melting (SLM)-manufactured 316 stainless steel and the influence of scanning speed. Tawfik et al. [16] investigated the effects of scanning speed on the microstructural characteristics and tensile behaviors of the Al-Mg aluminum alloy.

The influence of the AM scan pattern on residual stress has also been examined. Cheng and coworkers [17] analyzed the residual stresses of SLM-manufactured Ti-6Al-4V and In718. Song et al. [18] examined the influence of scanning patterns on Ti-6Al-4V parts using the FE numerical model. Sun et al. [19] investigated the effects of scanning patterns on the residual stress of aluminum alloys fabricated by AM. They considered the different scanning patterns, such as raster, zig-zag, in–out, out–in spiral, and alternate. Somashekara and coworkers [6] examined the influence of scan patterns on twin-wire welding-based AM. According to their numerical calculations and experimental measurements, raster scan patterns are recommended for TWAM, but the authors pointed out that the accuracy of the numerical model can be further improved by taking into account phase transformations and adopting the thermo-elastoplastic behaviors of the material.

In the present study, the effects of the AM scan pattern and speed on the residual stress in WAAM are examined using finite element simulations. The constitutive equation proposed by Leblond and Devaux [20] is applied to the stress update process using the ABAQUS user subroutines in order to consider the phase transformation and transformation plasticity in AM. The phase evolution is also examined in the present FE model, calibrated with various cooling rates in a CCT diagram. Analyses of the thermal history and phase volume fractions are conducted to establish the relationship between the AM process parameters and the product reliability.

## 2. Materials and Methods

### 2.1. Thermal and Metallurgical Analysis

The phase evolution equation proposed by Leblond and Devaux [20] is implemented into the present FE model with the energy equation to take into account the phase transformation in WAAM, as shown in Equation (1):

(1) $${\dot{p}}_{i}=-\sum_{j=1,j\neq i}^{N}A_{ij}(T,\dot{T}),\;\;i=1,2,\ldots ,N,$$

(2) $$\sum_{i=1}^{4}p_{i}=1\;\mathrm{for}\;t>0,$$

where $p_{i}$ is the phase volume fraction and $A_{ij}(T,\dot{T})$ is the rate of phase transformation from *j* to *i*. The details of $A_{ij}(T,\dot{T})$ are discussed in Section 2.3.

For the martensitic phase transformation, the model proposed by Koistinen et al. [21] is used. In this model, the phase volume fraction of the martensite can be expressed as follows:

(3) $$\begin{array}{c} p_{3}(T)=1-\exp\left\{a\left(T_{3,S}-T\right)\right\}\;\left(T\leq T_{3,S}\right)\\ with\;a=-1.10\times 10^{-2} \end{array},$$

where $T_{3,S}$ is the start temperature for the transformation from austenite to martensite.

The energy equation, taking into account the phase transformation, is expressed in Equation (4). The boundary conditions are imposed on the surfaces where the heat source is applied (*S_q_*) and where convective and radiative heat transfer (*S_θ_*) occur, as shown in Equations (5) and (6), respectively.

(4) $$\sum_{i}p_{i}(\rho c{)}_{i}\frac{dT}{dt}+\sum_{i}{\dot{p}}_{i}\rho_{i}H_{i}=\nabla \cdot \left(\sum_{i}p_{i}\lambda_{i}\nabla T\right),$$

(5) $$\left(-\right)\lambda \frac{\partial T}{\partial n}=q\;\mathrm{on}\;S_{q},$$

(6) $$\left(-\right)\lambda \frac{\partial T}{\partial n}=\chi_{1}\left(T-T_{0}\right)+\chi_{2}\left(T^{4}-T_{0}^{4}\right)\;\mathrm{on}\;S_{\theta },$$

where $\rho_{i},\;H_{i},c_{i},\;\lambda_{i}$ are the density, enthalpy, specific heat capacity, and thermal conductivity of phase i, respectively. $T_{0}$ is the ambient temperature (*T_0_* = 20 °C) and $\chi_{1}$ = 2.0 × 10^−5^ W/mm^2^ and $\chi_{2}$ = 2.82 × 10^−13^ W/mm^2^ are the convective and radiative heat transfer coefficients, respectively.

### 2.2. Heat Source Model for WAAM

The double ellipsoidal model proposed by Goldak et al. [22] has been extensively used to simulate the heat source for arc welding and, similarly, for the WAAM. This heat source model is defined with the help of two ellipsoids, the front and the rear, that are defined by the equations

(7) $$Q_{f}=\frac{6\sqrt{3}f_{f}Q}{{ab}_{f}c\pi \sqrt{\pi }}\exp\left(-\frac{3x^{2}}{a^{2}}-\frac{3y^{2}}{{b_{f}}^{2}}-\frac{3z^{2}}{c^{2}}\right),$$

(8) $$Q_{r}=\frac{6\sqrt{3}f_{r}Q}{{ab}_{r}c\pi \sqrt{\pi }}\exp\left(-\frac{3x^{2}}{a^{2}}-\frac{3y^{2}}{{b_{r}}^{2}}-\frac{3z^{2}}{c^{2}}\right).$$

Here x, y, and z are the coordinates of the heat source; $b_{f}$ and $b_{r}$ are parameters that represent the front and rear size of the heat source, respectively; and *a* and *c* represent the width and depth of the heat source, respectively. *Q* is the heat source energy. The values of the parameters for the double ellipsoidal model used in this work are listed in Table 1. Those values were chosen from Hammad et al. [23], which were validated by comparison with experimental measurements.

### 2.3. Calibration with CCT Diagram for Phase Evolution Analysis

Since phase transformation affects both thermal and mechanical behaviors of WAAM parts, calibration with a CCT diagram was carried out to perform the metallurgical analysis accurately. In Equation (1), the term $A_{ij}(T,\dot{T})$ represents the transformation from phase i to phase j ($A_{ij}$ > 0) or vice versa ($A_{ij}$ < 0) [20]. It can be further elaborated as Equation (9):

(9) $$A_{ij}=\left\{\begin{array}{l} k_{ij}(T)p_{i}-l_{ij}(T)p_{j}\\ if\;k_{ij}(T)p_{i}-l_{ij}(T)p_{j}>0(i\to j\;transf.)\\ -k_{ji}(T)p_{j}+l_{ji}(T)p_{i}\\ if\;k_{ji}(T)p_{j}-l_{ji}(T)p_{i}>0(j\to i\;transf.)\\ 0\;if\;k_{ij}(T)p_{i}-l_{ij}(T)p_{j}\leq 0\\ and\;k_{ji}(T)p_{j}-l_{ji}(T)p_{i}\leq 0\\ \left(no\;transformation\;between\;phases\;i\;and\;j\right) \end{array}.\right.$$

Following the suggestion of Leblond and Devaux [20], they are defined as follows:

(10) $$k_{ij}=\frac{P_{eq}(T)}{\tau },l_{ij}=\frac{1-P_{eq}(T)}{\tau }.$$

Here, $P_{eq}(T)$ is the proportion of equilibrium after an infinitely long time. τ denotes the time necessary for an equilibrium state. The value of $P_{eq}(T)$ is determined by consideration of the start and finishing temperatures ($T_{s}$ and $T_{f})\;$ of phase transformations and can be defined as:

(11) $$P_{eq}\left(T\right)=\frac{T_{s}-T}{T_{s}-T_{f}}.$$

The rate of phase change may be defined as Equation (11):

(12) $$\frac{dP}{dt}=\frac{P_{eq}-P}{\tau }.$$

Application of Taylor’s expansion to Equation (12) offers us phases $(P_{n+1}$) at time $t_{n+1}$ as follows:

(13) $$P_{n+1}=P_{n}+{\left.\frac{dP}{dt}\right|}_{n}{\left(\frac{dT}{dt}\right)}^{-1}\Delta T=P_{n}+\frac{P_{eq}-P}{\tau }{\left(\frac{dT}{dt}\right)}^{-1}\Delta T.$$

Here, $\frac{dT}{dt}$ is the cooling rate, while ΔT denotes the temperature difference between $t_{n+1}$ and $t_{n}$. Computation was conducted iteratively for the calibration with the CCT curves such that *T_f_* was in agreement with the values in the CCT diagram. Table 2 shows the parameters, τ obtained from the calibration for ferritic and banitic transformation. We note that Equation (3) is employed for martensitic transformation in this work.

**Figure 1:**
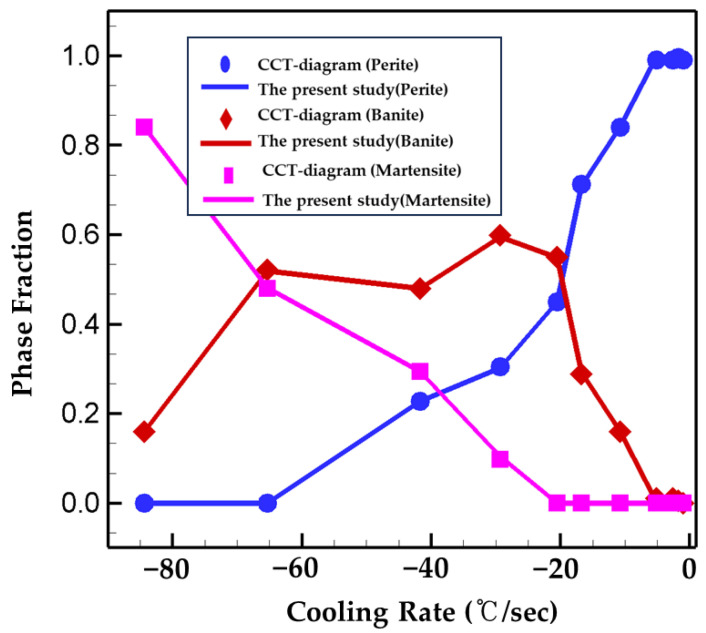
Verification of the phase fraction calculation of the present numerical model.

In order to consider the influence of phase transformation in WAAM procedure, Equations (1) and (9) are implemented into the present numerical model with the metallurgical parameters, obtained from calibration with the CCT diagram of EH36 steel. The computation results of phase volume fraction using the calibrated parameters (τ) are plotted with the ones in the CCT diagram for the purpose of verification of the present model. The calculations are in good agreement with the ones in the CCT diagram for the different cooling rates considered as shown in Figure 1.

### 2.4. Mechanical Analysis

The thermo-elastoplastic formulation is implemented to the present numerical model which takes into account phase transformation and transformation plasticity proposed by Leblond et al. [24,25,26,27]. The total strain ($\varepsilon^{tot}$) is decomposed of the elastic strain ($\varepsilon^{el}$), the thermo-metallurgical strain ($\varepsilon^{thm}$), the strain due to transformation plasticity ($\varepsilon^{tp}$), and the strain due to conventional plasticity ($\varepsilon^{cp}$) as in Equation (14):

(14) $$\varepsilon^{tot}=\varepsilon^{el}+\varepsilon^{thm}+\varepsilon^{tp}+\varepsilon^{cp},$$

where $\varepsilon^{thm}$ is given by

(15) $$\varepsilon^{thm}=(1-z)\varepsilon_{1}^{th}(T)+z\varepsilon_{2}^{th}(T).$$

Following Leblond et al. [20], the phase mixture of steel consists of two phases (i.e., the weak phase (γ) or austenite and the hard phase (α) consisting of bainite, ferrite and, martensite). $\varepsilon_{1}^{th}$ and $\varepsilon_{2}^{th}$ are the thermal strains of α and γ phases, respectively. z is the phase volume fraction of the α-phase.

The average yield strength of the hard phase can be calculated as the summation of each ferritic phase as shown in Equation (16):

(16) $$\sigma_{2}^{y}\left(T\right)=\sum_{i}p_{i}\sigma_{i}^{y},$$

where $p_{i}$ is the phase volume fraction of each phase, while $\sigma_{i}^{y}$ is the yield strength of each phase i. The nonlinear mixture rule is employed to determine the yield stress of the AM products and is given by

(17) $$\sigma^{y}\left(\varepsilon_{1}^{eff},\varepsilon_{2}^{eff},T\right)=[1-f(z)]\sigma_{1}^{y}\left(T,\varepsilon_{1}^{eff}\right)+f(z)\sigma_{2}^{y}\left(T,\varepsilon_{2}^{eff}\right).$$

Here, $\sigma_{1}^{y}$, $\sigma_{2}^{y}$, f(z) is the yield stresses of the hard phase, the weak phase and the modification factor, respectively.

In the present study, the plastic deformation is categorized with two regimes: the transformation plasticity ($\bar{\sigma }\leq \sigma^{y}$) and the macroscopic plasticity ($\bar{\sigma }=\sigma^{y}$). The constitutive model for the stress update is based on the hypoelastic formulation. It is first assumed that the AM part undergoes elastic deformation (i.e., ${∆\varepsilon }^{pl}$ = 0). Hence, the trial stress is expressed as in Equation (18):

(18) $$\sigma_{n+1}^{\mathrm{trial}\;}=\sigma_{n}+C^{el}:\Delta {\hat{\varepsilon }}^{RN}+\Delta C:\varepsilon_{n}^{el}.$$

Plastic deformation is categorized into two cases as described above: transformation plasticity ($\bar{\sigma }\leq \sigma^{y}$, occurs in cooling process) and macroscopic plasticity ($\bar{\sigma }=\sigma^{y}$). If the AM part undergoes plastic deformation, plastic relaxation is calculated by using the radial return mapping scheme in a different way depending on whether the type of deformation is transformation plasticity or conventional macroscopic plasticity. Such plastic relaxation is plugged into the constitutive equations to satisfy the yield criterion. Interested readers could find more details in references [26,27].

## 3. Results and Discussions

Thermo-mechanical simulations were utilized to establish the relationship of performance with process parameters. This study utilized the finite element (FE) method to examine the impact of AM scan pattern and speed on temperature field and residual stress as the process parameters.

The durability and structural performance of AM products are highly affected by their thermal profile, including temperature distribution and cooling rate. The thermal history of AM deposition is significantly affected by the amount of input heat and the heat transfer to the environment. The present study focuses on the effects of the AM scan pattern and speed on thermal history changes and residual stresses.

Due to its exceptional properties at low temperatures, EH36 is widely utilized in the maritime industry. EH36 steel is chosen for its potential application in WAAM. The material properties used in the simulations are listed in Table 3 [28]. The latent heat is 251,400 J/Kg and the solidus and liquidus temperatures are 1465.1 °C and 1522.5 °C, respectively.

**Figure 2:**
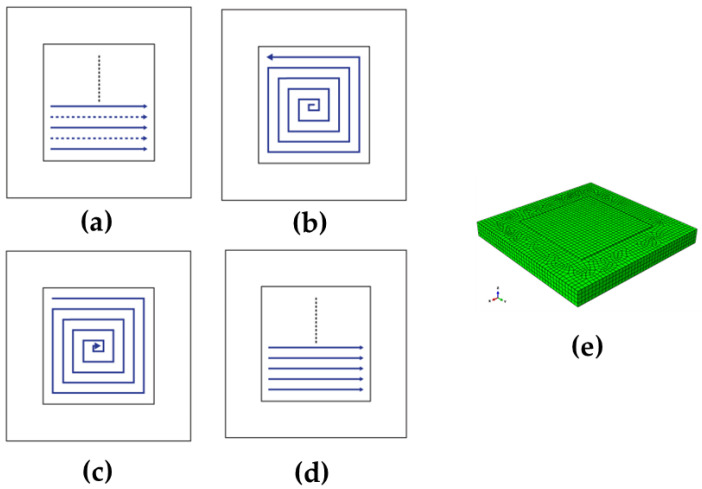
Deposition patterns analyzed in the present study. (**a**) Alternate, (**b**) in–out, (**c**) out–in, and (**d**) raster and (**e**) the FE numerical model in this work.

In the present study, four scan patterns (alternate, in–out, out–in, and raster as shown in Figure 2) and three cases for the AM scan speed of 25 cm/min, 40 cm/min and 50 cm/min are considered to examine their effects on residual stress and warpage. The substrate has the dimensions of 350 mm × 350 mm × 30 mm discretized with 32,764 elements. A sequentially coupled thermo-mechanical analysis is conducted using the ABAQUS user subroutines UMAT. Eight-node linear heat-transfer brick elements, DC3D8, are used for thermal analyses, while C3D8 eight-node linear brick elements are used for mechanical simulations. Temperature distribution and phase transformation are first examined for the different scan patterns and speeds. Afterward, analyses on how such process parameters affect the production of residual stress are performed.

### 3.1. The Scanning Pattern

**Figure 3:**
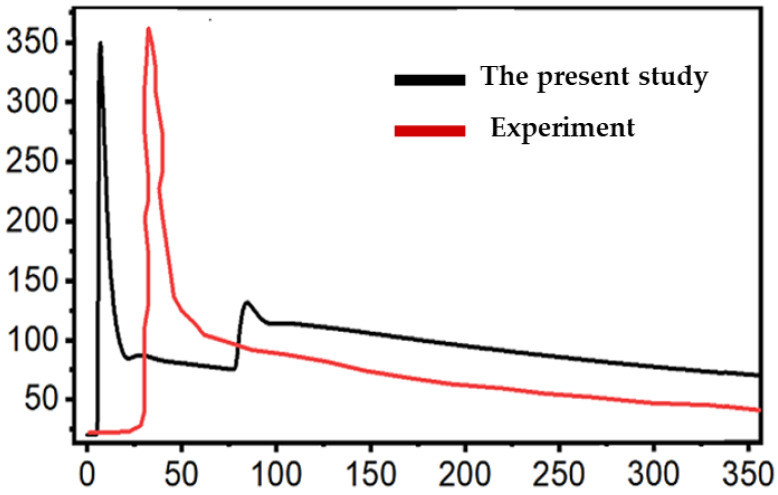
Verification of the thermal analysis of the present numerical model.

Figure 3 shows the comparison of temperature calculations with the experiment measurements of Ding et al. [29] for the verification of the present numerical model. The alternate scanning case was chosen, and the investigation point was located 5 mm away from the path of deposition, following the experiment setup of Ding et al. As shown in Figure 3, the values of peak temperature are similar for both cases. Overall, the trend of temperature curves for both cases are in good agreement with each other. The slight discrepancy of the temperature profiles may result from the different cooling rates due to the welding travel speed and/or boundary conditions.

**Figure 4:**
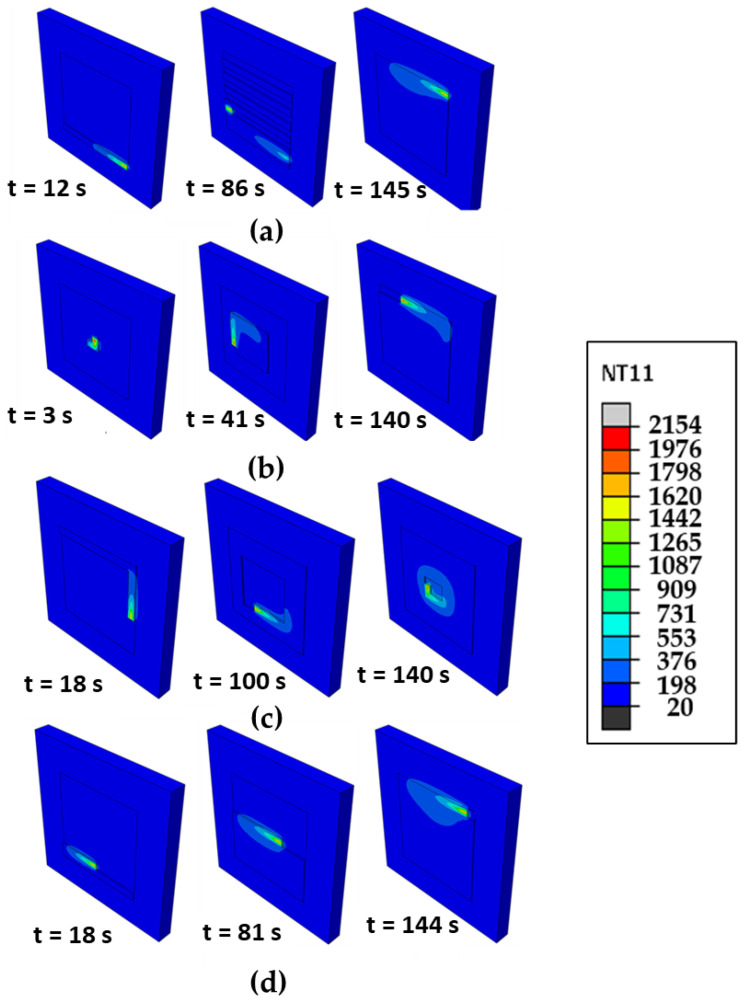
Time evolution of temperature distribution for different scanning patterns (Unit: °C): (**a**) alternate, (**b**) in–out, (**c**) out–in, and (**d**) raster.

**Figure 5:**
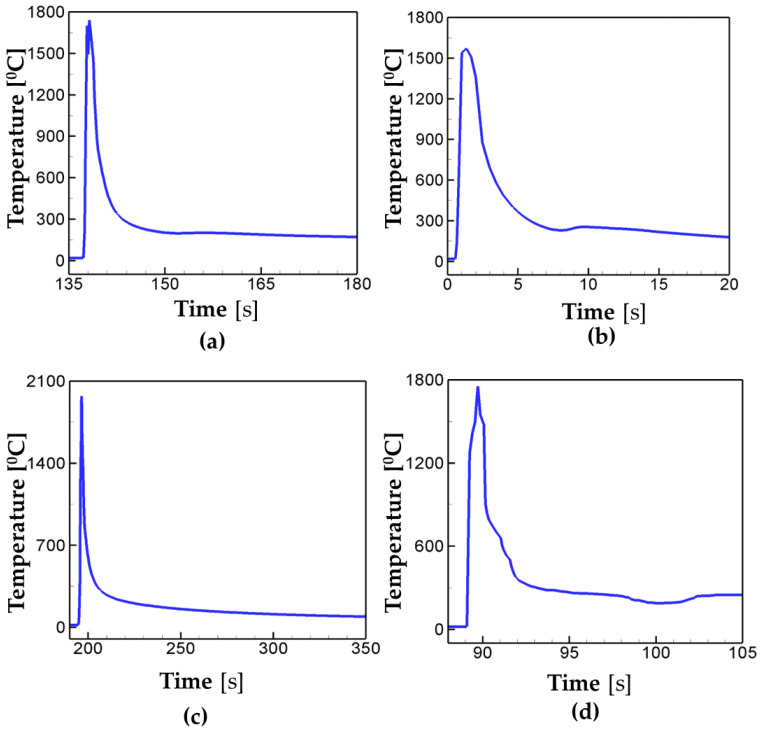
Temperature profile at the center of the deposition layer for the different scan patterns: (**a**) alternate, (**b**) in–out, (**c**) out–in, (**d**) raster.

Figure 4 shows the time evolution of temperature distribution for the different scanning patterns. The alternate and raster patterns are discontinuous, requiring multiple starts and stops. The in–out and out–in patterns follow spiral contours, which require fewer passes but produce excessive thermal gradients. In the case of the out–in pattern, the heat energy is more concentrated than in other cases due to the characteristics of its trajectory. This results in a higher value of peak temperature than that of other cases as shown in Figure 5, which is addressed later. We note that in case of the alternate pattern, it has the lowest level of temperature contour as well as the smallest size of the heat-affected zone among the scanning pattern considered. This is mainly attributed to the fact that the alternate pattern has an interval between each scanning, which allows rapid heat transfer to the surroundings. We note that the raster pattern is also the line-path scanning the same as the alternate pattern. However, it does not have intervals between each deposition. Such characteristic leads to a higher thermal gradient and a wider HAZ compared with the alternate case. Analyses so far suggest that the scanning pattern significantly affects the thermal profiles such as thermal gradient, cooling rate, and peak temperature. This could eventually affect the formation of residual stress and warpage. To examine this issue further, the evolutions of temperature and phase volume fraction are analyzed.

Figure 5 shows the temperature profiles for the alternate, in–out spiral, out–in spiral, and raster pattern at the center of the deposition. In Figure 5a, the peak temperature for the case of alternate scanning drops to below 300 °C faster than any other cases considered. The cooling speed of the raster (Figure 5d) comes second, while the in–out and out–in cases show the slow cooling rate. The alternate scanning pattern has the longest distance between each deposition pass among the cases considered in the present study. This is attributed to the fastest cooling rate in the case of the alternate scan. It is worth noting that the in–out and out–in scanning patterns have continuous scanning paths, and the heat is accumulated due to their path characteristics which lead to the slower cooling rate than in the discontinuous scanning cases such as the alternate and the raster. In case of the in–out pattern (Figure 5b), the peak temperature is around 1500 °C, which is 300 °C lower than the ones for other cases. This is mainly because the in–out pattern starts its scanning at the center (i.e., at the point of the investigation), different from the other cases, and thus it does not have time for heat to transfer to the center as the scanning proceeds. The findings so far suggest that the AM scan pattern could have great influence on the cooling rate of deposition, leading to the change in structural properties. 

**Figure 6:**
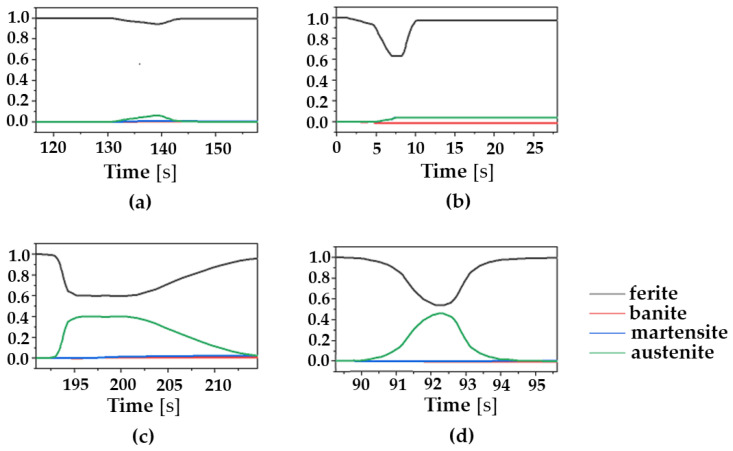
Phase volume fraction at the center of the deposition layer for the different scanning patterns: (**a**) alternate, (**b**) in–out, (**c**) out–in, and (**d**) raster.

Next, the effects of the AM scan pattern on phase transformation are investigated. Ferritic steel (ferrite/pearite, banite, or martensite) has a bcc structure at room temperature, while it transforms into austenite above the eutectoid temperature (T = 715 °C). In cooling after AM deposition, austenite transforms into ferritic steels and their volume fractions are different depending on the cooling rate. Figure 6 shows the phase transformation at the center point of a deposition layer for the different scanning patterns. Figure 5 and Figure 6 show that the ferritic steel starts to transform into austenite when the temperature exceeds the eutectoid temperature during the heating cycle for all cases considered. For all cases considered in the present study, the phase fraction of ferrite/pearlite is calculated to be greater than 95%. This major formation of pearlite at the end of AM deposition illustrates that the cooling rate is not high enough to nucleate martensite. The phase evolution curves in Figure 6 suggest that the AM scan pattern could lead to different phase transformation, because it is dependent on thermal history and cooling rate.

**Figure 7:**
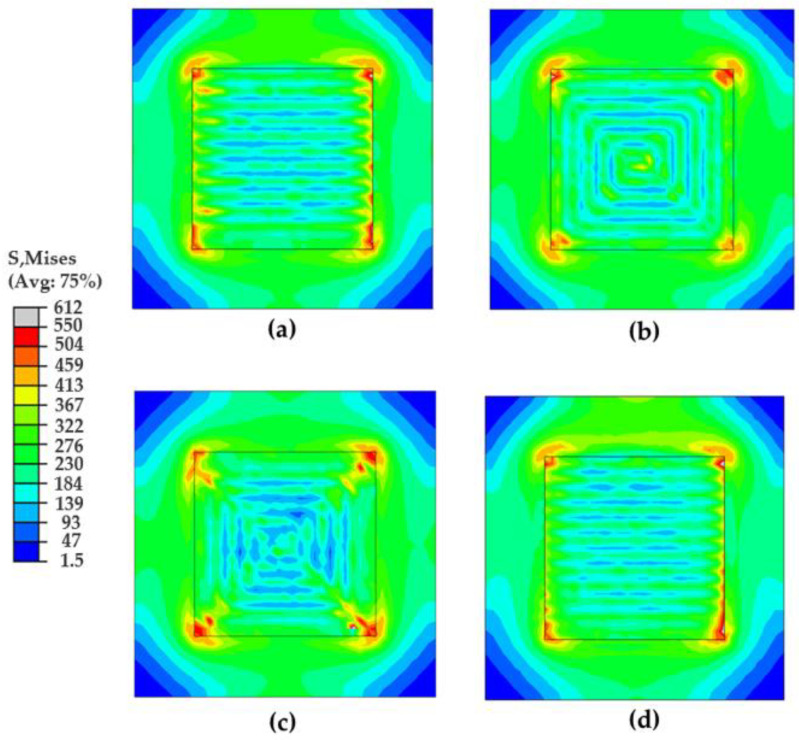
Mises stresses distribution at the end of simulations for the different scan patterns (Unit: MPa): (**a**) alternate, (**b**) in–out, (**c**) out–in, and (**d**) raster.

Figure 7 shows the Mises stress distribution at the end of the simulation for the different scanning patterns. The highest stress is concentrated at the corner of the deposition layer, while the lowest one is located at the corner of the substrate. For all cases considered, high values of stress are generated along the deposition lines following the scanning path, while the low values are located between the deposition paths.

**Figure 8:**
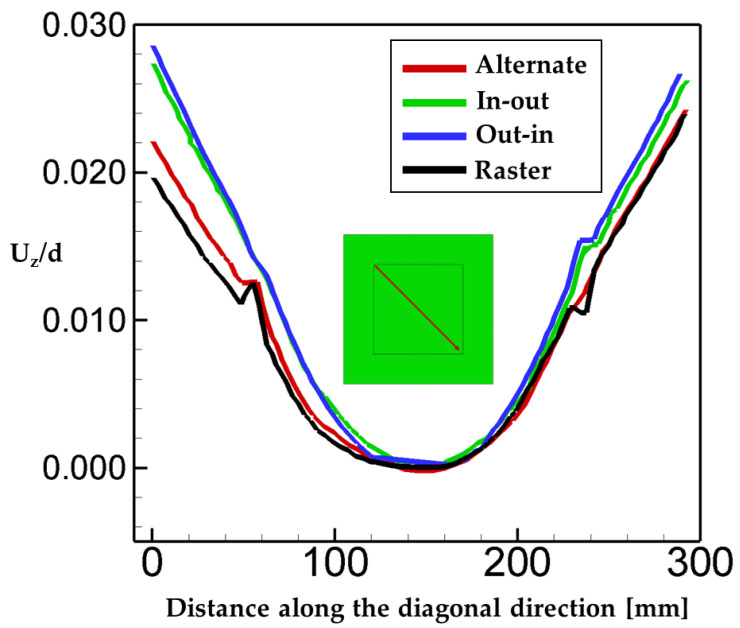
Warpage for the different scan patterns (normalized by substrate thickness).

Warpage can cause excessive distortion, and thus should be taken into account to optimize the AM process. In the present study, the deposition is built up in the z-direction. The deformation in the z-direction of the deposition layer is analyzed and plotted in Figure 8 as a function of distance along the diagonal direction. We note that the displacement in the z-direction is normalized by the thickness of the substrate. The scanning patterns considered in the present study can be categorized into the line type (alternate and raster) and the spiral type (in–out and out–in). It is interesting to note in Figure 8 that the deflection in the case of spiral types is greater than the one in the case of line types. That is mainly attributed to the fact that spiral-type patterns have continuous paths due to their characteristic of the scanning path which induces the accumulation of heat in deposition. On the contrary, the trajectory of line-type patterns is discontinuous, leading to lesser heat accumulation in deposition.

**Figure 9:**
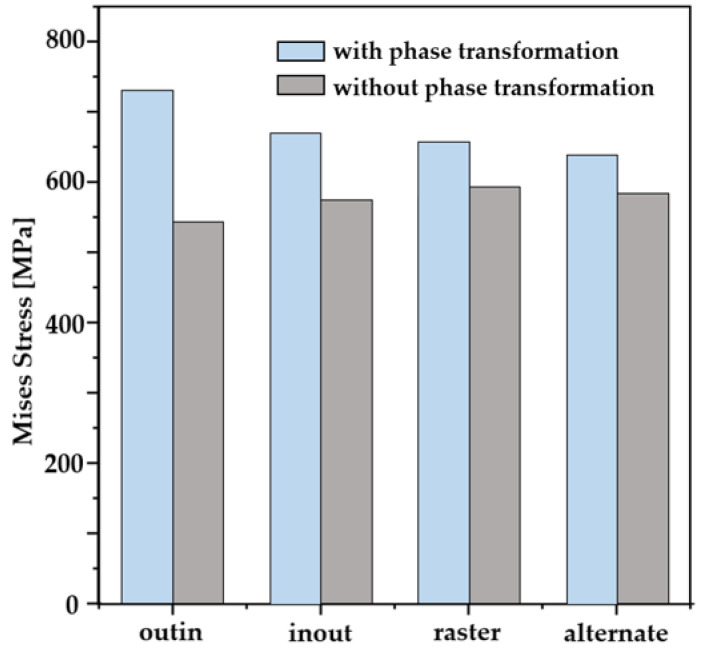
Maximum Mises stress for the different scanning patterns.

In order to investigate the residual stress and the correlation between the scanning pattern and phase transformation, the maximum values of Mises stresses are examined for the different scan patterns with and without consideration of phase transformation. These results are compared and plotted in Figure 9. As shown in the Figure 9, higher values of Mises stress are generated in the case of phase transformation considered than the ones with no phase transformation considered. Another interesting finding is that the difference in the maximum Mises stresses between with and without consideration of phase transformation is greater in the case of continuous scanning patterns (i.e., the out–in (189 MPa); the in–out (86 MPa)) than in discontinuous ones (the raster (67 Mpa); the alternate (46 Mpa)). We note that in the case of continuous scanning patterns, the heat is accumulated due to their path characteristics, which leads to the slow cooling rate. It is also worth noting that the out–in pattern has the longest time of austenitic phase transformation among all the cases. It also has the highest portion of the austenite volume fraction as shown in Figure 6c. Such results suggest that the cooling rate can significantly differ depending on the choice of the AM scanning pattern, and this affects the phase transformation and residual stress generation in the AM products. Among the AM process parameters, the scanning speed can also affect the cooling rate of the deposition. This issue is examined in the Section 3.2.

### 3.2. Effect of AM Scanning Speed

In this section, the effects of scanning speed on thermal profiles such as cooling rate, temperature, and phase transformation are examined to unveil the mechanism of residual stress generation. Finite element simulations of AM deposition are conducted with three different scanning speeds: 25 cm/min, 40 cm/min and 50 cm/min. The in–out pattern is chosen for such analysis.

**Figure 10:**
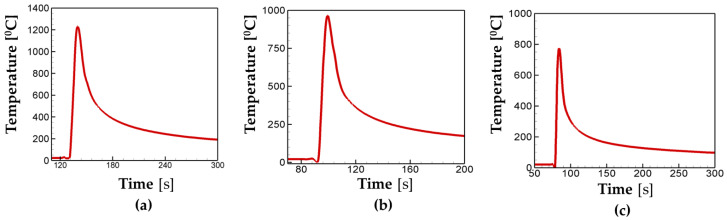
Temperature history for the different scan speeds of (**a**) 25 cm/min, (**b**) 40 cm/min, and (**c**) 50 cm/min.

Figure 10 shows the temperature evolution for the different scanning speeds. In the case of 25 cm/min, peak temperature is calculated as T = 1200 °C, and then it drops to below 200 °C 165 s after it reaches its maximum. It is worth noting that in Figure 5, the choice of scanning pattern hardly changes the peak temperature except in the in–out case due to its path characteristics. However, in Figure 10, the peak temperature decreases with an increase in scanning speed. The peak temperature is calculated as T = 975 °C and T = 782 °C for the scanning speeds of 40 cm/min and 50 cm/min, respectively. Analyses in Figure 10 demonstrate that the faster the scanning speed applied to the system, the less time is available for heat accumulation, which results in low peak temperature. In addition, the value of thermal conductivity of steel decreases with an increase in temperature, which decreases the heat transfer performance. That can explain why the fast scan speed leads to a lower peak temperature and a faster cooling rate.

**Figure 11:**
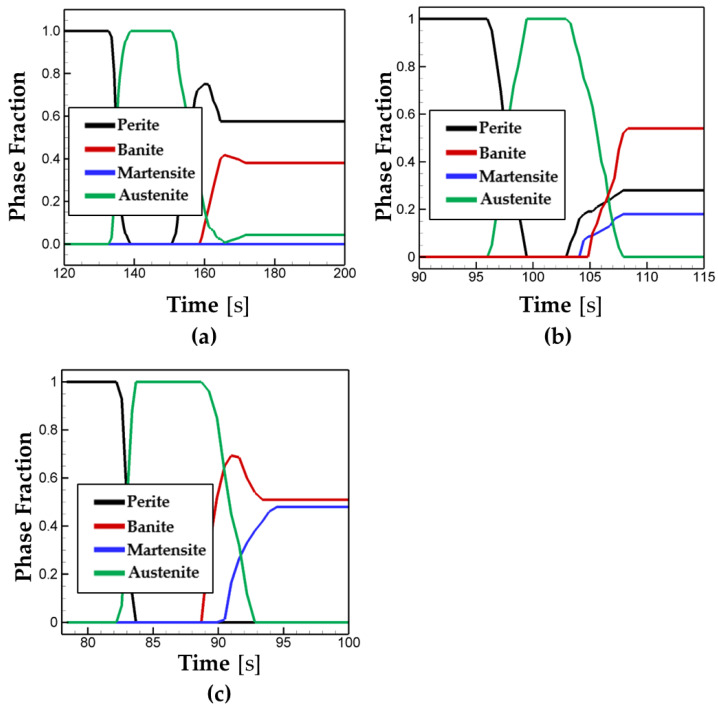
The phase fraction for the different scanning speeds of (**a**) 25 cm/min, (**b**) 40 cm/min, and (**c**) 50 cm/min.

Next, the values of the phase volume fraction are compared and plotted in Figure 11 for different scanning speeds. In the case of the 25 cm/min scanning speed, the major portion of the phase fraction is calculated as pearlite (56%) and banite (35%), and no martensite is formed. In the cases of the higher scan speed, it is observed that the martensite is formed, and its phase portion increases with an increase in scanning speed as shown in Figure 11b,c.

**Figure 12:**
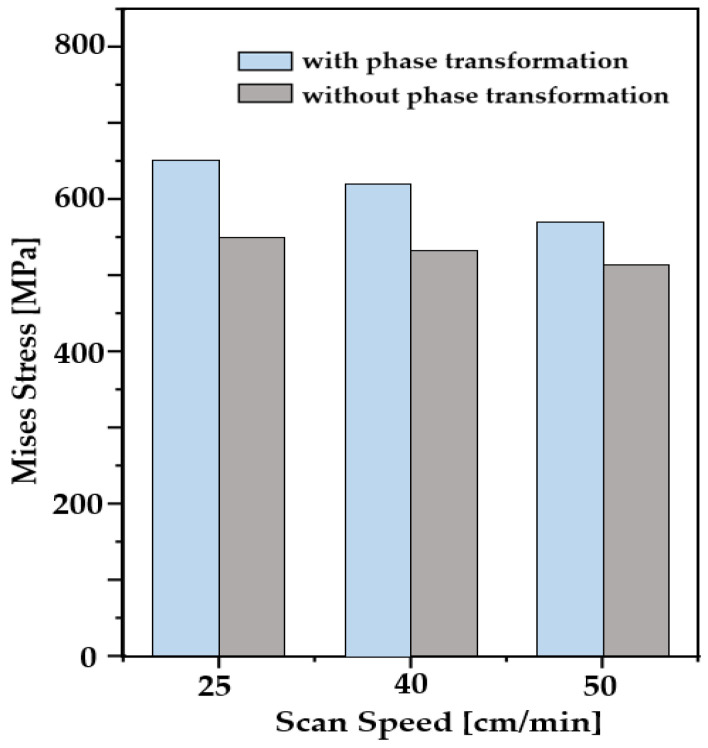
Maximum Mises stress for the different scanning speeds.

In order to establish the correlation among scan speed, cooling rate, and residual stress generation in the AM process, the values of maximum Mises stresses are compared and plotted in Figure 12 with and without consideration of the phase transformation for the different scan speeds. Overall, the generation of residual stress in AM decreases with an increase in scan speed. In addition, the effects of phase transformation on residual stress also decreased as scan speed increased. It is expected that the influence of phase transformation on residual stress generation to be reduced and become insignificant as scan speed increases further.

## 4. Conclusions

In the present study, WAAM simulations were performed with consideration of phase transformation. The effects of the scan pattern and the speed on temperature, phase transformation, residual stress, and warpage were examined. Four scanning patterns and three deposition speeds were investigated. Major findings are the follows:

1. Results show that out–in scanning generates the highest value of residual stress (732 MPa), while alternate scanning leads to the lowest residual stress (627 MPa) among the cases considered.
2. It is found that the choice of scanning pattern hardly changes the peak temperature, while the amount of heat accumulation during the deposition and the cooling rate are significantly dependent on the type of scanning pattern. It leads to different phase volume fractions and various levels of residual stresses.
3. The difference in maximum Mises stresses between the cases with and without consideration of phase transformation is also compared for different scanning patterns. In the case of continuous scanning patterns, the heat is accumulated due to its path characteristics, which leads to a slow cooling rate and allows the longest time of austenitic phase transformation.
4. Analyses show that the scanning speed changes both the level of peak temperature and the cooling rate. It is found that an increase in scanning speed leads to a decrease in residual stress.
5. In the case of the spiral scanning pattern, the value of deflection is greater than the one in line-type patterns. That is mainly attributed to the fact that the spiral-type patterns have continuous paths due to their characteristic of scanning paths which induce heat accumulation in deposition and lead to a slow cooling rate.

The present study considered the influence of scanning patterns and speeds on thermal profiles, residual stress, and warpage in single layer deposition. The heat source energy and the idle time between each deposition could also affect the thermal gradient and cooling rate. A study on the effects of such process parameters on structural reliability of AM products is underway. The numerical model and parametric study in the present work can be employed in other types of processes such as PBF AM or arc welding.

## Figures and Tables

**Table 1:** The heat source parameters in Equations (7) and (8).

$b_{f}$ (mm)	$b_{r}$ (mm)	a (mm)	c (mm)	$f_{f}$	$f_{r}$	Q (W)
5	10	3.35	5	0.6	1.4	7258

**Table 2:** Parameter τ for phase transformation in cooling.

Cooling Rate (°C/s)	Ferritic Transformation			Banitic Transformation		
	T_s_ (°C)	T_f_ (°C)	τ (s)	T_s_ (°C)	T_f_ (°C)	τ (s)
−0.98	699.8	581.2	0.95	581.2	508.0	0.001
−1.74	689.0	570.4	0.001	570.4	501.2	0.001
−2.58	678.9	567.8	0.01	567.8	494.2	0.001
−5.08	651.2	562.2	0.007	562.2	484.0	0.001
−10.71	624.1	555.5	0.9775	555.5	461.7	0.005
−16.72	612.7	545.8	1.05	545.8	446.3	0.01
−20.46	603.3	550.1	1.95	550.1	424.2	0.001
−29.35	587.8	546.7	1.8	546.7	454.3	0.44
−41.67	567.9	543.7	1.055	543.7	452.0	0.91
−65.29	No ferritic transformation occurs			537.0	448.9	0.787
−84.39				526.4	451.1	1.82

**Table 3:** Material properties of EH36 steel [28].

Temperature (°C)	Thermal Conductivity (W/(m°C))	Density (kg/m^3^)	Young’s Modulus (GPa)	Thermal Expansion Coefficient (10^−6^/°C)	Specific Heat (J/g°C)	Poisson Ratio
25	45.87	7851	208.6	12.48	0.435	0.289
100	45.83	7837	204.3	12.88	0.454	0.292
200	45.08	7813	199.5	12.90	0.497	0.294
300	43.13	7755	189.9	13.37	0.554	0.300
400	40.83	7726	181.6	13.89	0.605	0.307
500	37.88	7696	168.4	14.36	0.660	0.308
600	35.13	7664	156.3	13.65	0.768	0.311
700	32.50	7622	136.8	13.86	0.962	0.317
800	29.55	7599	125.6	15.26	0.920	0.327
900	27.65	7560	112.8	15.44	0.651	0.347
1000	28.87	7531	102.6	15.65	0.605	0.354
1100	30.05	7500	92.2	15.74	0.624	0.359
1200	31.37	7452	83.0	16.05	0.624	0.364
1300	32.55	7394	43.1	16.32	0.647	0.370
1400	33.69	7340		16.62	0.663	0.378

## Data Availability

The data that support the findings of this study are available from the corresponding author upon reasonable request.
